# Lattice-patterned LC-polymer composites containing various nanoparticles as additives

**DOI:** 10.1186/1556-276X-7-46

**Published:** 2012-01-05

**Authors:** Kyoseung Sim, Shi-Joon Sung, Eun-Ae Jung, Dae-Ho Son, Dae-Hwan Kim, Jin-Kyu Kang, Kuk Young Cho

**Affiliations:** 1 Green Energy Research Division, DGIST, 50-1 Sang-ri, Hyeonpung-myeon, Dalseong-gun, Daegu, 711-873, Republic of Korea; 2 Division of Advanced Materials Engineering, Kongju National University, 275 Budae-dong, Cheonan, Chungnam, 331-717, Republic of Korea

**Keywords:** phase separation, nanoparticle, LC-polymer composite, photopolymerization, lattice pattern.

## Abstract

In this study, we show the effect of various nanoparticle additives on phase separation behavior of a lattice-patterned liquid crystal [LC]-polymer composite system and on interfacial properties between the LC and polymer. Lattice-patterned LC-polymer composites were fabricated by exposing to UV light a mixture of a prepolymer, an LC, and SiO_2 _nanoparticles positioned under a patterned photomask. This resulted in the formation of an LC and prepolymer region through phase separation. We found that the incorporation of SiO_2 _nanoparticles significantly affected the electro-optical properties of the lattice-patterned LC-polymer composites. This effect is a fundamental characteristic of flexible displays. The electro-optical properties depend on the size and surface functional groups of the SiO_2 _nanoparticles. Compared with untreated pristine SiO_2 _nanoparticles, which adversely affect the performance of LC molecules surrounded by polymer walls, SiO_2 _nanoparticles with surface functional groups were found to improve the electro-optical properties of the lattice-patterned LC-polymer composites by increasing the quantity of SiO_2 _nanoparticles. The surface functional groups of the SiO_2 _nanoparticles were closely related to the distribution of SiO_2 _nanoparticles in the LC-polymer composites, and they influenced the electro-optical properties of the LC molecules. It is clear from our work that the introduction of nanoparticles into a lattice-patterned LC-polymer composite provides a method for controlling and improving the composite's electro-optical properties. This technique can be used to produce flexible substrates for various flexible electronic devices.

## Introduction

Owing to its impact on device performance, the phase separation behavior of materials and its effect on the device morphology have attracted considerable attention as one of the powerful methods for fabricating flexible electronic devices, such as organic photovoltaics, organic field effect transistors, organic nonvolatile memory devices, and liquid crystal displays [LCDs] [1-6]. The phase separation of a mixture is attributed to the difference in surface free energy among the components and their interactions with each other. Lattice-patterned liquid crystal [LC]-polymer composites, which are characterized by phase separation of the mixture of LC and the miscible photoreactive monomers upon UV light irradiation under a patterned mask, are one of the most important fabrication materials for flexible substrates that can be used in flexible electronics, owing to their sophisticated and controllable non-contact characteristics [7,8].

As the region of the mixture that is irradiated by UV light undergoes a photoreaction to form polymerized polymer walls that act as a supporting structure, the monomer and LC simultaneously diffuse into polymer-rich and polymer-poor regions, respectively, through dynamic phase separation. This is the cause of the difference in the surface free energy and the low miscibility between the LC molecules and the UV-cured polymers. The phase separation can be used to determine the features of cells containing the LC surrounded by polymer walls. These structures are resistant to bending stress, satisfying a fundamental requirement of flexible electronic substrates.

However, as in all organic material systems, the control of physical and electro-optical properties of LC-polymer composites is limited due to the restricted properties of the organic materials. Nowadays, in order to overcome the limitations of all organic material systems, many research groups have become interested in enhancing phase separation using hybrid materials, which involves introducing inorganic materials into the system. To minimize the deterioration of the display properties, such as the transparency, it is preferable to use inorganic materials in the form of nanoparticles as additives [9-13].

In this study, we show the effects of introducing inorganic nanoparticles into lattice-patterned LC-polymer composites on the phase separation behavior and electro-optical properties of the composites. Prepolymers containing nanoparticles were prepared by mixing UV-curable monomers and SiO_2 _nanoparticles of varying sizes and with various surface functional groups. Photoinduced phase separation was caused by exposing the LC-prepolymer mixtures to UV light by using a lattice-patterned photomask. The phase separation structures of the lattice-patterned LC-polymer composites were then studied using polarized optical microscope imaging, and the electro-optical properties of the LC were investigated by measuring the contrast ratio and the driving voltage of the lattice-patterned LC-polymer composites.

## Experimental details

**Figure 1:**
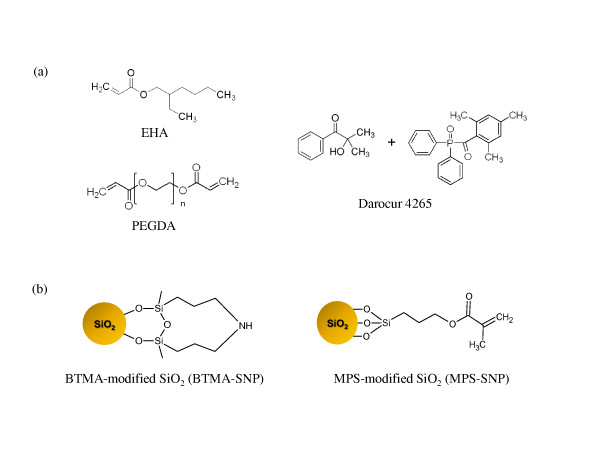
**Chemical structures of the components for prepolymers**. (**a**) EHA, PEGDA, and Darocur 4265 used for the preparation of prepolymers and (**b**) functionalized SiO_2 _nanoparticles with BTMA and MPS.

A UV-curable prepolymer solution was prepared by mixing ethylhexyl acrylate [EHA] (Sigma-Aldrich Corporation, St. Louis, MO, USA; used as a monomer), polyethyleneglycol diacrylate [PEGDA] (Sigma-Aldrich Corporation, St. Louis, MO, USA; used as a cross-linker), and Darocur 4285 (Sigma-Aldrich Corporation, St. Louis, MO, USA; used as a photoinitiator); Figure 1a shows the chemical structures of these compounds. In order to investigate the effects of particle size and surface functional groups, four different types of SiO_2 _nanoparticles were synthesized. SiO_2 _nanoparticles with diameters of 7 and 14 nm that were not subjected to post-treatment are denoted by SiNP-7 and SiNP-14, respectively. Two different types of functionalized SiO_2 _nanoparticles were prepared using bis[3-(trimethoxysilyl)propyl]amine [BTMA] and [3-(methacryloyloxy)propyl] trimethoxy silane [MPS] based on silane coupling reactions, and they were labeled BTMA-SNP and MPS-SNP, respectively (Figure 1b). The quantities of SiO_2 _nanoparticles were 1, 3, and 5 wt.% relative to the weight of the acrylate prepolymer solutions.

Lattice-patterned LC-polymer composites were prepared by exposing the mixture containing the prepolymer solution (50 wt.%) and LC (E7, Merck KGaA, Darmstadt, Germany; 50 wt.%) to UV light. The LC cell was prepared using two ITO glass slides coated with rubbed polyimide, between which the LC-prepolymer mixture was injected by capillary effect at 100°C, which was above the clearing temperature. To form the polymer walls, UV light was radiated for 400 s through a photomask comprising 300 × 300 μm^2 ^of dark square patterns with a 30-μm spacing. The sample temperature was maintained at 100°C. Polarized optical microscope images of the lattice-patterned LC-polymer composites were obtained, and the polymer wall thickness was determined by using a microscope system (Eclipse LV100D, Nikon Co., Shinjuku, Tokyo, Japan). In order to confirm the effect of the various SiO_2 _nanoparticles on the electro-optical properties of the LC, the contrast ratio and the driving voltage of the LC-polymer composites were measured.

## Results and discussion

**Figure 2:**
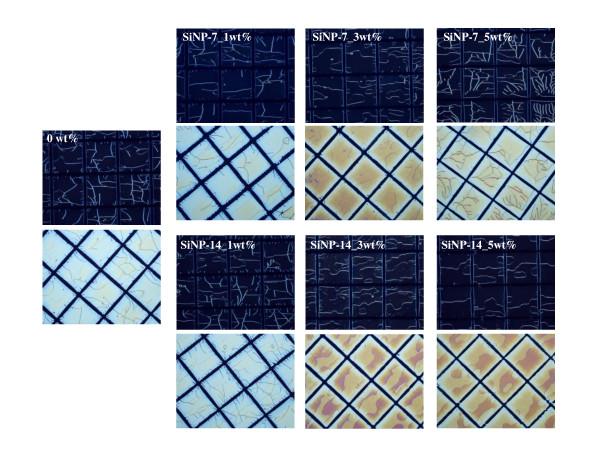
**Polarized optical microscope images of lattice-patterned LC-polymer composites containing non-functionalized SiO_2 _nanoparticles as additives**.

**Figure 3:**
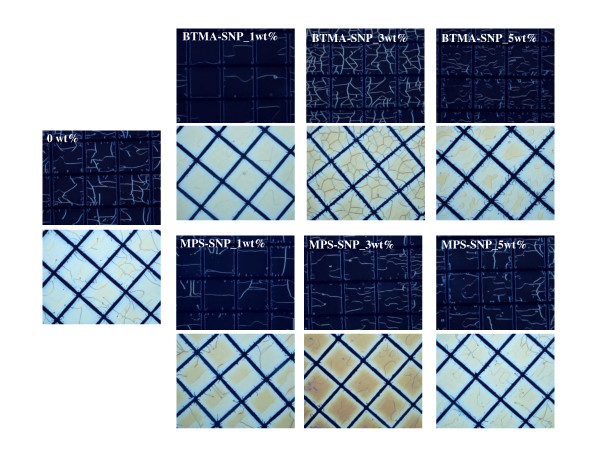
**Polarized optical microscope images of lattice-patterned LC-polymer composites containing functionalized SiO_2 _nanoparticles as additives**.

**Figure 4:**
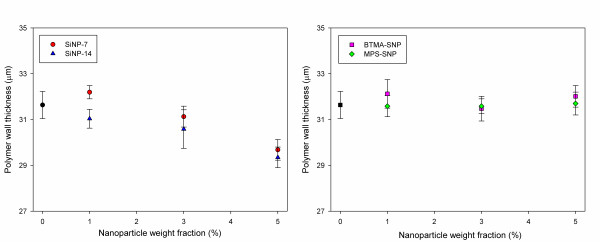
**The polymer wall thickness of lattice-patterned LC-polymer composites containing non-functionalized and functionalized SiO_2 _nanoparticles**.

Figure 2 presents polarized optical microscope images of the lattice-patterned LC-polymer composites prepared by using mixtures of the LC and UV-curable prepolymers containing untreated SiO_2 _nanoparticles (SiNP-7 and SiNP-14) as an additive. All the images show cells of LC molecules surrounded by UV-cured polymer walls. From the polarized optical microscope images, it can be seen that the dependence of the phase separation structure on the quantities of SiNP-7 and SiNP-14 is small. The polymer wall thickness decreased slightly with an increase in the quantities of SiNP-7 and SiNP-14. In the cases of the functionalized SiO_2 _nanoparticles, BTMA-SNP and MPS-SNP, the LC-polymer composites also showed a similar phase separation structure regardless of the quantities of BTMA-SNP and MPS-SNP (Figure 3). However, the polymer wall thickness did not change with the quantities of BTMA-SNP and MPS-SNP. The wall thickness reduction in the lattice-patterned LC-polymer composites with an increase in the quantities of SiNP-7 and SiNP-14 could be interpreted as being due to untreated SiO_2 _nanoparticles diffusing in the direction opposite to that of the polymerizing regions [14,15]. Thus, SiNP-7 and SiNP-14 migrated into the LC domains during photoinduced phase separation, resulting in a loss of volume for SiNP-7 and SiNP-14 and a loss in their free volume in the polymeric region. The reduction in the volume of the polymer walls corresponded to an increase in the quantities of SiNP-7 and SiNP-14, and thus, the polymer wall thickness also decreased. However, in the case of the functionalized SiO_2 _nanoparticles, the presence of alkyl chains gave the nanoparticles a hydrophobic character, which resulted in the particles being sequestered within the polymeric region [15]. BTMA-SNP and MPS-SNP were expected to remain in the polymeric region during photoinduced phase separation, and the volume of the polymer walls containing BTMA-SNP or MPS-SNP should not have changed with the quantities of BTMA-SNP and MPS-SNP. Therefore, there was no change in the polymer wall thickness regardless of the quantity of the surface-treated inorganic nanoparticles. Figure 4 summarizes the changes in the polymer wall thickness with the size and surface functional groups of the SiO_2 _nanoparticles. As can be clearly seen in the polarized optical microscope images, the polymer wall thickness decreased with an increase in the quantities of SiNP-7 and SiNP-14, whereas no difference was observed for the cases of BTMA-SNP and MPS-SNP. Thus, the functional groups of SiO_2 _nanoparticles were closely related to the phase separation behavior of the lattice-patterned LC-polymer composites, and the polymer wall thickness was affected only by the inclusion of untreated SiO_2 _nanoparticles.

**Figure 5:**
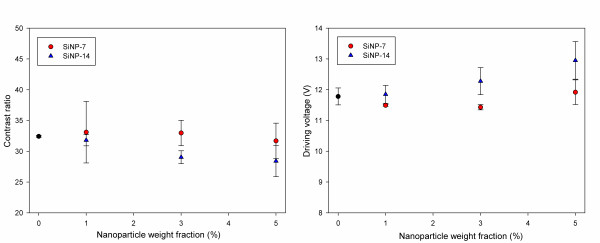
**Electro-optical properties of lattice-patterned LC-polymer composites containing non-functionalized SiO_2 _nanoparticles as additives**. The contrast ratio for a driving voltage of 12.5 V and the driving voltage corresponding to a contrast ratio of 30 in the case of lattice-patterned LC-polymer composites containing non-functionalized SiO_2 _nanoparticles as additives.

In addition to the phase separation behavior of the LC-polymer composites, it is important to investigate the electro-optical properties of the composites when SiO_2 _nanoparticles are included in the prepolymer systems from the viewpoint of the applications of the composites. To this end, we measured the contrast ratio and driving voltage of the lattice-patterned LC-polymer composites. The contrast ratio of the LC is the ratio of light transmittance between the on-state and the off-state of the LC, and the driving voltage is the intensity of external electric field required to obtain the contrast ratio of the LC. The contrast ratio and driving voltage are known to affect the electro-optical properties of conventional LCDs. Figure 5 illustrates the contrast ratio and driving voltage of the LC-polymer composites containing SiNP-7 or SiNP-14. The contrast ratio decreased with the quantities of SiNP-7 and SiNP-14, and the range over which the contrast ratio decreased was more apparent for SiNP-14. When an external electric field was applied to the composites, the LC molecules rotated. In terms of driving the LC molecules, the SiO_2 _nanoparticles can hinder the movement of LC molecules, and thus, the contrast ratio of the LC would decrease with an increase in the quantity of SiO_2 _nanoparticles. These results also support the view that the untreated SiO_2 _nanoparticles, SiNP-7 and SiNP-14, were mainly located in the LC regions after photoinduced phase separation. In the case of the driving voltage, the SiO_2 _nanoparticles can have a similar hindrance effect on the movement of the LC molecules, and thus, the driving voltage of the LC molecules would increase with an increase in the quantity of SiO_2 _nanoparticles. In the case of SiNP-14, SiO_2 _nanoparticles with larger diameters can disturb the movement of the LC molecules more easily than those of SiNP-7, and thus, the changes in the contrast ratio and driving voltage were more evident than in the case of SiNP-7.

**Figure 6:**
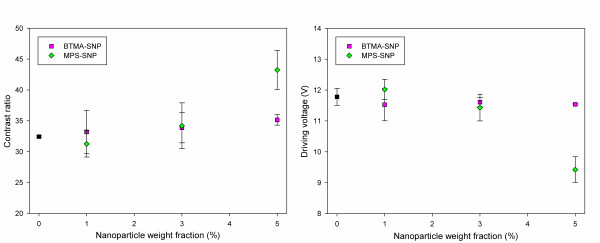
**Electro-optical properties of lattice-patterned LC-polymer composites containing functionalized SiO_2 _nanoparticles as additives**. The contrast ratio for a driving voltage of 12.5 V and the driving voltage corresponding to a contrast ratio of 30 in the case of lattice-patterned LC-polymer composites containing functionalized SiO_2 _nanoparticles as additives.

In contrast with the case of untreated SiO_2 _nanoparticles, BTMA-SNP and MPS-SNP had different impacts on the electro-optical properties of the lattice-patterned LC-polymer composites (Figure 6). In the case of BTMA-SNP, the contrast ratio of the LC increased from 32 to 35, and the driving voltage remained about 11.5 V regardless of the quantity of BTMA-SNP. The surface of the BTMA-SNP was covered with a hydrophobic BTMA functional group and thus maintained its location in the polymeric region after photoinduced phase separation. It is thought that the surface region of the polymer walls may have been affected by the hydrophobic BTMA-SNP and that the interaction between the LC and the polymer walls could thus have been weakened by the BTMA-SNP. Owing to the decreased interaction between the LC and the polymer walls, the movement of the LC molecules upon the application of an external electric field was less likely to be hindered by the polymer walls, and the electro-optical properties could have been improved by the inclusion of BTMA-SNP in the prepolymers. However, BTMA-SNP would aggregate in the polymer walls because the nanoparticle-polymer interactions were unfavorable with respect to polymer-polymer interactions. The entropy of mixing of the polymeric and nanoparticle systems is generally quite small, and thus, even small unfavorable polymer-nanoparticle interactions can lead to nanoparticle aggregation. Therefore, no improvement in the electro-optical properties of the LC-polymer composites upon the inclusion of BTMA-SNP was evident.

In the case of MPS-SNP, the positive effect of SiO_2 _nanoparticles on the electro-optical properties was more evident than in the case of BTMA-SNP. The contrast ratio of the LC increased from 32 to 43, and the driving voltage decreased to about 9.5 V as the quantity of MPS-SNP increased to 5 wt.%. MPS-SNP nanoparticles were modified by using an MPS functional group, which had a long alkyl chain and a methacryloyl group, for the photopolymerization reaction. Similar to BTMA-SNP, MPS-SNP also shows hydrophobic properties because of its long alkyl chains and is thus expected to remain in the polymeric region during photoinduced phase separation. However, compared with BTMA-SNP, MPS-SNP has a photoreactive methacryloyl functional group and can also participate in the photopolymerization of prepolymers. Therefore, MPS-SNP can be uniformly distributed in the region around the polymer walls after photoinduced phase separation and can prevent the aggregation of MPS-SNP in the polymer walls. Owing to the uniform distribution of MPS-SNP in the polymer walls, the surface region of the walls might have been well covered by MPS-SNP, and thus, the improvement in the electro-optical properties of the LC-polymer composites was more evident than it was for BTMA-SNP.

**Figure 7:**
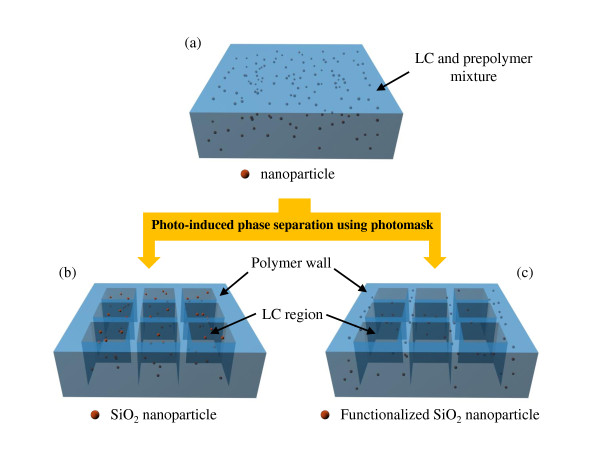
**Schematic diagram of the distribution of SiO_2 _nanoparticles in lattice-patterned LC-polymer composites**. (**a**) The mixture of an LC, a prepolymer, and SiO_2 _nanoparticles, (**b**) the distribution of non-functionalized SiO_2 _nanoparticles, and (**c**) the distribution of functionalized SiO_2 _nanoparticles after photoinduced phase separation.

Figure 7 shows a schematic diagram of the distribution of SiO_2 _nanoparticles in the lattice-patterned LC-polymer composites. The starting mixture contained an LC, a prepolymer, and nanoparticles as additives (Figure 7a). When the LC-prepolymer mixtures with nanoparticles were irradiated by UV light through a photomask with dark square patterns, polymerization occurred in the irradiated area. As a result, the mixture was divided into polymer walls (the polymer region) and cells (the LC region) by photoinduced phase separation between the LC and the prepolymer. Subsequently, the lattice-patterned LC-polymer composites were formed. If we had added nanoparticles to the LC-prepolymer mixture system, the position of the nanoparticles would have changed from the LC region to the polymeric region, depending on the surface functional groups of the nanoparticles. Because of the hydroxyl group on their surface, the non-functionalized SiO_2 _nanoparticles diffused into the LC region during photoinduced phase separation and remained in the LC region (Figure 7b). The SiO_2 _nanoparticles in the LC regions might have hindered the movement of the LC molecules, and thus, a change in the distribution of the nanoparticles from the LC region to the polymeric region was required. On the other hand, Figure 7c shows the case of functionalized SiO_2 _nanoparticles in the prepolymer systems. In this case, the surface region of the SiO_2 _nanoparticles was covered by functional groups, such as BTMA and MPS. When the alkyl chains were used as a surface functional group, the surface properties of the SiO_2 _nanoparticles could be easily changed from hydrophilic to hydrophobic. Therefore, the functionalized SiO_2 _nanoparticles resided in the polymeric region after photoinduced phase separation, and they did not affect the LC region. The SiO_2 _nanoparticles in the polymeric region could have also contributed to the interfacial properties between the LC and the polymer walls. However, the hydrophobic SiO_2 _nanoparticles tended to aggregate in the polymeric region, and thus, the addition of SiO_2 _nanoparticles to the prepolymers was less effective for controlling the interfacial properties of the LC-polymer composites. To prevent the aggregation of SiO_2 _nanoparticles in the polymer walls, a surface functional group that could participate in photopolymerization would be required. SiO_2 _nanoparticles with MPS could participate in photopolymerization, and the SiO_2 _nanoparticles would be uniformly distributed in the polymer walls. Thus, the effects of SiO_2 _nanoparticles with MPS on electro-optical properties could be greater than those of SiO_2 _nanoparticles with BTMA.

## Conclusions

We found that phase separation behavior and interfacial properties of lattice-patterned LC-polymer composites were significantly affected by the inclusion of various SiO_2 _nanoparticles in the prepolymers. The distribution of SiO_2 _nanoparticles in the LC-polymer composites was affected by the surface functional groups of the nanoparticles. Untreated SiO_2 _nanoparticles were mainly located in the LC region, and thus, the behavior of the LC molecules was disturbed by these nanoparticles. Surface functionalized SiO_2 _nanoparticles remained in the polymeric region after photoinduced phase separation due to the hydrophobic alkyl chains of the surface functional groups. The SiO_2 _nanoparticles in the polymer walls were closely related to the interfacial properties between the LC and the polymer walls. In particular, SiO_2 _nanoparticles with a photoreactive methacryloyl group could participate in the photopolymerization of the prepolymers. Owing to the uniform distribution of the SiO_2 _nanoparticles with a methacryloyl group, the electro-optical properties of the LC-polymer composites were effectively improved by the inclusion of the nanoparticles. By using customized nanoparticles with various sizes and surface functional groups as additives, it might be possible to control the phase separation behavior and the interfacial properties of lattice-patterned LC-polymer composite systems. Such control can facilitate the use of the LC-polymer composite system for the fabrication of various flexible electronic devices.

## Competing interests

The authors declare that they have no competing interests.

## Authors' contributions

KS interpreted the experimental results and prepared the manuscript draft and figures. SJS proposed the main research idea, completed the final manuscript, and presided over the study. EAJ carried out most of the experiments. DHS operated the experimental instruments used in the study. DHK and JKK verified the data interpretation. KYC helped in revising the manuscript. All the authors read and approved the final manuscript.
